# Analysis of Erosive Nature of Fruit Beverages Fortified with Calcium Ions: An In Vitro Study Evaluating Dental Erosion in Primary Teeth

**DOI:** 10.1155/2022/3756384

**Published:** 2022-06-09

**Authors:** Priyanka Dedhia, Deepika Pai, Shyam Dutt Shukla, U. Anushree, Saurabh Kumar, Kalyana C Pentapati

**Affiliations:** ^1^Pediatric Dentist, The Smile Suite, Children and Family Dental Care, Mumbai, India; ^2^Associate Professor, Department of Pedodontics and Preventive Dentistry, Manipal College of Dental Sciences, Manipal Academy of Higher Education, Manipal 576104, India; ^3^Department of MTech, Research Assistant Machine Tools Laboratory, IIT, Mumbai, India; ^4^Department of Medical Biochemistry, Kasturba Medical College, Manipal Academy of Higher Education, Manipal 576104, India; ^5^Associate Professor, Department of Public Health Dentistry, Manipal College of Dental Sciences, Manipal Academy of Higher Education, Manipal 576104, India

## Abstract

**Background:**

Since children frequently consume acidic fruit beverages, dental erosion is common in children. The erosive effects on primary teeth are more profound due to the lesser thickness of enamel and dentin. This study evaluated if calcium fortification of fruit beverages could reduce their erosive potential. *Methodology*. Tropicana Orange Delight was the fruit beverage chosen and fortified with calcium carbonate. Forty noncarious extracted primary teeth were equally distributed into four groups. Samples in group A were exposed to calcium-fortified fruit beverages and group B to nonfortified fruit beverages for 1, 2, and 3 min. The pH and calcium ion concentration of both the fruit beverages were evaluated from baseline through the test period. Samples in groups C and D were then exposed to fortified and nonfortified fruit beverages for 10, 20, and 30 min. The surface roughness and microhardness of these samples were analysed.

**Results:**

Due to fortification, the pH and calcium ion concentration of fortified beverages was higher compared to nonfortified beverages at baseline. The calcium ion concentration of fortified beverages decreased from baseline in contrast to an increase in the nonfortified beverage group. This indicates that fortified beverage is less erosive in nature. The surface roughness of samples in the fortified beverage group did not decrease significantly from baseline. In the nonfortified beverage group, surface roughness values at 20 and 30 min of exposure were higher than baseline, suggesting that significant erosive changes occur at the prolonged duration of exposure. The microhardness values of samples in the fortified beverage group increased from baseline through the test period, suggestive of resistance to erosion offered by calcium fortification. The microhardness values of samples in the nonfortified beverage group decreased through the test period, suggestive of erosion.

**Conclusion:**

Calcium fortification of this fruit beverage significantly reduces its erosive potential on primary teeth.

## 1. Introduction

Dental erosion results from the loss of hard tissue of the tooth, including enamel, dentin, and cementum, primarily caused by increased consumption of acidic beverages. The severity of erosion depends on the acidic nature of the beverage consumed, frequency of consumption, the mineral composition of the tooth, the protective factors from saliva, and so on [1]. Fruit beverages being an easy source of hydration and nutrition are frequently consumed by children. Hence, dental erosion is commonly seen in children. A review of the literature on the prevalence of dental erosion in young children reveals that approximately 15–59% of children demonstrate dental erosion [2–5]. An increasing trend of higher prevalence of erosion in the preschool children of developed and developing countries has also been reported [6]. Few studies showed that 32%–90% of children between the age group of 2–7 years exhibit dental erosion [7, 8]. Since consumption of fruit beverages cannot be avoided in children, dental erosion is seen as an inevitable sequela. Therefore, many researchers have attempted to reduce the erosive potential of these beverages.

The addition of hydrocolloids, magnesium, calcium citrate malate, fluoride, and calcium/phosphate to the soft beverages and fruit beverages was observed to significantly reduce their erosive potential [9]. Some studies showed that high amounts of calcium, phosphate, and fluoride in therapeutic agents can reduce the erosion of enamel [10, 11].

The pH buffering capacity and ionic composition of the beverages particularly the concentration of calcium and phosphate can largely influence their erosive potential [11, 12]. Amongst the most effective way to chemically modify the erosive potential of a beverage, the supplementation of the beverage with soluble calcium salts is considered to be the best established method [13]. We conducted this study to evaluate the effect of calcium ion fortification on the erosive potential of the fruit beverage on primary teeth. In our study, Tropicana Orange Delight®, a favourite and commonly available packaged fruit beverage consumed by children, was fortified using powdered calcium carbonate. This study aimed to compare the erosive potential of a calcium-fortified fruit beverage to the same beverage without calcium fortification on primary teeth. After exposing the teeth samples of all groups to the experimental fruit beverages, the changes in pH, calcium ion concentration of the fruit beverages, surface roughness, and microhardness of the teeth samples were evaluated. The changes in pH and calcium ion concentration of the fruit juices correlated to the influence of fortification on the erosive nature of the beverage instantly. The changes in surface roughness and microhardness of teeth samples were assessed after exposing the samples for 10, 20, and 30 min to the beverages. This was done to evaluate the influence of prolonged exposure to beverage on the teeth as seen in abnormal consumption patterns like continuous sipping of the beverage, holding of the beverage in the mouth for an extended time before swallowing as seen commonly in children [14, 15].

A null hypothesis was proposed that there is no difference in the erosive potential of fortified and nonfortified fruit beverages on primary teeth.

## 2. Materials and Methods

### 2.1. Study Design

This in vitro study was conducted after obtaining institutional ethical committee approval (IEC 707/2014).

Forty extracted primary teeth with intact coronal tooth structure were indicated for extraction due to exfoliative mobility or orthodontic treatment needs. Primary teeth with white spot lesions, fractured enamel surfaces, restorations, and hypoplastic defects were excluded from the study. The primary teeth indicated for extraction were stored in 0.1% thymol until sample preparation and in deionised water until the beginning of the test. The samples were distributed into four groups (*n* = 10). The samples in group A were exposed to the fortified fruit beverage, and samples in group B were exposed to the nonfortified fruit beverage for 1, 2, and 3 min. The pH and calcium ion concentration of the fruit beverage at baseline and the end of the test period were recorded. The samples in groups C and D were exposed to fortified and nonfortified fruit beverages for 10, 20, and 30 min, respectively. The surface roughness and microhardness of these teeth samples from baseline through the test period were evaluated.

### 2.2. Sample Preparation

The primary teeth fulfilling the inclusion criteria were stored in 0.1% thymol solution. The enamel specimens were prepared by sectioning the crown from the root of the tooth using a diamond disk at high speed and air-water spray. The crown portion of the sample was then sectioned longitudinally along the mesiodistal dimensions to obtain an intact labial/buccal surface. The sectioned surfaces were then embedded in the acrylic resin blocks exposing the labial/buccal enamel surfaces.

Acid-resistant nail varnish was painted on the enamel surfaces. Windows measuring 6 mm by 4 mm, meant for immersion into the fruit beverages were exposed. The exposure window was further divided into three windows each measuring 2 mm by 4 mm surfaces to facilitate different exposure times in the same specimen. The right side of the teeth corresponding to the right window was colour-coded as pink. The left side of the teeth corresponding to the left window was colour coded as blue leaving uncoloured central part of the tooth corresponding to the central exposure window. The other section of the teeth sample was used to make baseline measurements.

### 2.3. Characterisation of Beverages

Measurement of pH: The pH was measured using a pH metre (Eutech instruments pH 700). The pH electrode was calibrated at the start of each session using standard buffers of pH 4.0 and pH 7.0 and was rinsed thoroughly between uses to avoid contamination. One litre of the newly opened beverage was placed in a beaker and stirred at a rate of 875 rpm until a stable reading was obtained and was considered as the baseline pH. Three readings were taken to give a mean measurement of the pH of that drink [16].

Fortification of fruit beverage: The buffering capacity of the fruit beverage was measured by placing 20 ml of the beverage in the glass beaker, and 1 N of sodium hydroxide was gradually pipetted until the pH was neutral. The volume of sodium hydroxide required to increase the pH of 1 litre of fruit beverage to neutral pH was recorded. Powdered calcium carbonate was added to fortify the beverage with calcium to obtain the concentration of 1 mmol/L of calcium ions [9]. The total amount of calcium in the beverage was determined spectrophotometrically at a wavelength of 575 nm (Thermo Scientific™ GENESYS 10 S UV-Vis spectrophotometer). Three sets of readings were taken to get the mean calcium ion concentration of the fortified fruit beverage [9]. In the present study, CaCO_3_ was used to fortify the fruit beverage, which has been approved by the World Health Organization for the fortification of food products [17].

### 2.4. Exposure of Tooth Samples to Fruit Beverages for pH and Calcium Ion Concentration Assessment

Baseline pH and calcium ion concentration of both the fortified and nonfortified fruit beverages were recorded. The samples were immersed in artificial saliva and left undisturbed for a period of 60 min before the start of the experiment. The artificial saliva contained the following composition: 150 mmol/L KCl, 1.5 mmol/L CaCl_2_, and 0.9 mmol/L KH_2_PO_4_ in 100 mL of distilled water (pH was adjusted to 7.0). During the evaluation of changes in pH and calcium ion concentration, the right half of the specimen was exposed to respective fruit beverages to be tested for 1 min; during this period, the other windows were covered with adhesive tape. The central window was exposed to fruit beverages for 2 min, while the exposure windows on right and left sides were covered with adhesive tape. The left experimental window was exposed to fortified and nonfortified fruit beverage groups for 3 min exposure, and the other windows were now covered with adhesive tapes. The samples in groups A and B were immersed entirely into the respective fruit beverages for the designated time. The samples were rinsed with deionised water before the next exposure. After each exposure, the pH and calcium levels of the fruit beverages were assessed.

### 2.5. Exposure of Tooth Samples to Fruit Beverages for Evaluating Surface Roughness and Microhardness

The baseline readings were recorded in samples without an exposure window. In groups C and D, samples with window corresponding to colour pink were immersed in fortified and nonfortified fruit beverages, respectively, for 10 min. The other exposure windows were taped during this period; a similar method was followed when other allocated windows were exposed to experimental beverages. The central exposure window was exposed to fortified and nonfortified fruit beverages in respective groups for 20 min. The left-sided blue coded window was exposed for 30 min in both groups C and D. The surface profile and microhardness were tested in all the exposure windows using an optical profilometer and a Vickers hardness tester [9, 12].

### 2.6. Surface Profilometry

Using an optical profilometer (ZETA 3D PROFILOMETER), the surface profile in each window of the sample was scanned. Five-line scans were performed in the centre of each sample at intervals of 100 *μ*m. Each sample was then assessed for Ra (arithmetic average of all deviations of the profile from the centreline) and Rz (mean of five roughness depths of five successive sample lengths of the profile) values. The step size was set at 0.01, and the number of steps was set at 450. The results of five scans were averaged for each sample, and the mean value was calculated and expressed in micrometres [18].

### 2.7. Surface Microhardness

The microhardness was tested in each window using a Vickers hardness tester (Shimadzu Vickers hardness tester) with a load of 100 g for 10 s as recommended by Davidson et al. [17]. Three indentations were made in each window, and the mean value was calculated and expressed as HV/10 [19, 20].

### 2.8. Statistical Analysis

The statistical analysis was performed using SPSS version 18. The pH, calcium ion concentration, surface roughness, and microhardness values were analysed using an independent sample *t*-test enabling intergroup comparison and repeated measures ANOVA with post hoc Bonferroni test enabling intragroup comparison. The level of statistical significance was set at *p* < 0.05.

## 3. Results

### 3.1. pH Changes in Fruit Beverages Analysed

The intergroup comparison of changes in pH showed that the baseline pH was higher in the fortified fruit beverage group than in the nonfortified fruit beverage group. The initial fall in pH from baseline to 1 min of exposure was significantly higher in the nonfortified fruit beverage group than the fortified fruit beverage group. The pH did not drop significantly thereafter in 2 and 3 min of exposure in both groups (Table 1).

In group A, there was an overall significant difference, in the mean scores from baseline through 3 min. The post hoc test showed that baseline had a significantly higher mean score than 1 and 3 min. Similarly, 2 min had a higher mean score than 3 min. No other significant differences were seen (Table 1).

Group B showed an overall significant difference, in the mean scores from baseline through 3 min. The post hoc test showed that baseline had a significantly higher mean score than 1, 2, and 3 min. No other significant differences were seen (Table 1).

The analysis of pH changes reveals that fortification of fruit beverages leads to a rise in baseline pH of the beverage, thereby bringing the pH to near neutral, making it less erosive.

### 3.2. Calcium Ion Concentration Changes of Fruit Beverages Analysed

The intergroup comparison of calcium ion concentration changes showed a significant difference in the level of baseline calcium ion concentration in the fortified fruit beverage group as compared to the nonfortified group attributed clearly to the calcium ion fortification. The total calcium ion concentration at 1, 2, and 3 min after exposure with respect to baseline depleted in fortified fruit beverages, whereas it increased in the nonfortified fruit beverage group (Table 1).

In group A, the post hoc test showed that baseline had a higher mean score of calcium ion concentration than 1, 2, and 3 min. The mean score of calcium ion concentration at 3 min was higher than 1 and 2 min after exposure (Table 1).

In group B, an overall significant difference in mean scores of calcium ion concentration from baseline through 3 min of exposure was observed. The post hoc test revealed that the mean score at 1 min of exposure was significantly higher than baseline and 2 and 3 min of exposure. The calcium ion concentration in group B (nonfortified fruit beverage) increased significantly from baseline to 1 min after exposure and then decreased after 2 and 3 min (Table 1) suggestive that peak movement of calcium ions takes place in the first 1 min of exposure.

### 3.3. Surface Profile Assessment of Samples Immersed in Fruit Beverages

The intergroup comparison of surface roughness showed that there was no significant difference in Ra and Rz values from baseline to 10, 20, and 30 min after exposure (Table 2).

**Figure 1:**
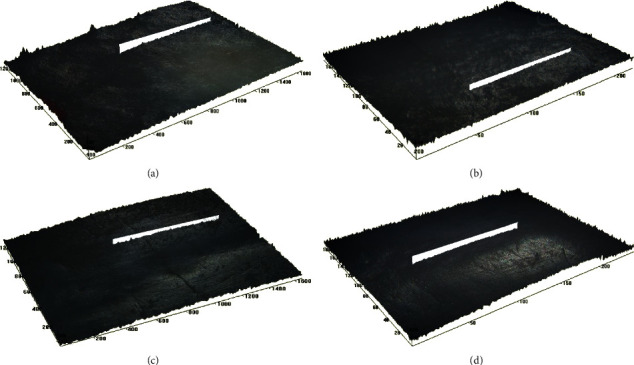
(a) Surface roughness of teeth sample from group C at baseline, under 10x magnification as observed under optical profilometer; (b) surface roughness of teeth sample from group C at 10 min after exposure to fortified fruit beverage, under 10x magnification as observed under optical profilometer; (c) surface roughness of teeth sample from group C at 20 min after exposure to fortified fruit beverage, under 10x magnification as observed under optical profilometer; and (d) surface roughness of teeth sample from group C at 30 min after exposure to fortified fruit beverage, under 10x magnification as observed under optical profilometer.

The Ra and Rz values (Table 2) of the samples in group C did not change significantly from baseline to 10, 20, and 30 min after exposure (Figure 1(a)–1(d)). The post hoc test showed the Rz had significantly higher mean scores at baseline than 20 and 30 min of exposure (Table 2).

**Figure 2:**
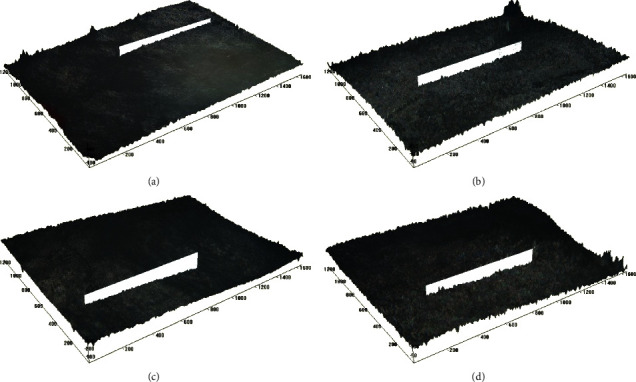
(a) Surface roughness of teeth sample from group D at baseline, under 10x magnification as observed under optical profilometer; (b) surface roughness of teeth sample 10 min after exposure to nonfortified fruit beverage (group D) at 10x magnification as observed under optical profilometer; (c) surface roughness of teeth sample 20 min after exposure to nonfortified fruit beverage (group D) at 10x magnification as observed under optical profilometer; and (d) surface roughness of teeth sample 30 min after exposure to nonfortified fruit beverage (group (D) at 10x magnification as observed under optical profilometer.

The Ra and Rz values of samples in group D showed an overall significant difference in mean scores from baseline to 10, 20, and 30 min after exposure (Table 2; Figure 2(a)–2(d)). The post hoc test showed that Ra values at 20 and 30 min had statistically higher mean scores than at baseline, and the Rz value at 30 min had significantly higher mean scores than baseline, 10 and 20 min (Table 2).

### 3.4. Surface Microhardness of Teeth Samples Immersed in Fruit Beverages

The intergroup comparison of microhardness values showed a significant increase from baseline to 10 min of exposure in group C as compared to group D. The microhardness value of samples in group C did not show a significant difference from 10 to 20 min after exposure as at 30 min from baseline. The samples in group D showed a significant reduction in the surface microhardness from baseline to 10, 20, and 30 min after exposure to nonfortified fruit beverages (Table 2).

In group C, there was an overall significant difference in mean score from baseline through the exposure of 30 min. The post hoc test showed that baseline had a significantly lower mean score than10, 20, and 30 min of exposure (Table 2).

In group D, there was an overall significant difference in the mean scores from baseline through 30 min of exposure. The post hoc test showed that baseline had a higher mean score than 10, 20, and 30 min. Also, the mean surface hardness score at 10 min was higher than 20 and 30 min, suggesting that the change in microhardness of samples varied at the duration of exposure (Table 2).

## 4. Discussion

Erosion is a measure of detrimental influence exerted by acidic substances including fruit or acidic beverages on the tooth. The interaction between the chemical composition and physical properties of the beverage consumed in the natural oral environment (biological factors) and individual consumption habits (behavioural factors) determine the severity of erosive changes [13].

The physicochemical properties of the beverage that strongly influence the rate of erosion include the pH, titratable acidity, buffering capacity, pKa of the acid, undissociated acid concentration, calcium chelating, and the degree of saturation (DS) of the drink [11, 13]. The degree of saturation of the beverage with respect to dental minerals (hydroxyapatite and fluorapatite) is determined by the Ca:P molar ratio, type of acid, calcium chelating properties, and undissociated acid concentration [13]. Some of these variables are interdependent, but all of them are known to influence the erosive potential of a given beverage. Hence, modifying a beverage can influence one or more of these variables and therefore collectively the erosion rate too [13].

In our study, the Tropicana Orange Delight was the fruit beverage tested that was the original/unfortified beverage. This beverage was then fortified with calcium carbonate powder; therefore, the study was aimed to evaluate the influence of the addition of calcium on the erosive potential of the beverage. The pH and calcium ion concentration of fruit beverages and surface roughness and microhardness of the teeth samples immersed in these fruit beverages were assessed as a measure of their erosive potential.

The pH of the fruit drink Tropicana Orange Delight that was chosen for the study was 3.92, after adding calcium carbonate to this beverage the pH increased to 6.96. Hence, the baseline pH of the unfortified fruit beverage was 3.92, and the baseline pH of the fortified fruit beverage was 6.96. The addition of calcium has resulted in a change in pH level as observed in our study. A study conducted by Franklin et al. on in vitro assessment of the erosive potential of calcium-fortified to calcium-nonfortified fruit beverages also has demonstrated that the addition of calcium to fruit beverage increases its pH [21].

The baseline pH of nonfortified fruit beverages used in our study was 3.93, which is acidic. The fortification of fruit beverages with calcium carbonate raised the pH to 6.96. The drop in pH of the calcium-fortified fruit beverage group from baseline to 1, 2, and 3 min after exposure was significantly less compared to the nonfortified fruit beverage group suggesting that the fortified fruit beverage is comparatively less erosive than nonfortified fruit beverage.

The pH of nonfortified fruit beverages reduced consistently and more significantly than the fortified fruit beverage group. These observations made from our study coincide with the results of the study conducted by Lussi and Jaeggi [22] and Seow and Thong [23].

The more acidic the pH, the higher the erosive potential of the beverage [17]. In our study, the baseline pH of fortified fruit beverage was as high as 6.96; it was observed to cause less erosion as opposed to its unfortified version where the pH was 3.92. Simply, the addition of calcium raised the pH of the fruit beverage, thereby rendering it less erosive than the unfortified beverage [21].

Reducing the acidity of the fruit beverage is an established method of reducing its erosive potential. Modification of beverages by increasing their pH (>3.8) and lowering titreable acidity has shown to have resulted in a considerable reduction of erosive potential [12]. Such results were also demonstrated by modified sports beverages with pH ranging from 5.5 to 5.6 proved to have a significantly weaker effect on hydroxyapatite dissolution compared to original beverages, the pH of which reaches 3.0 to 4.2 [24]. In our study, the addition of calcium influenced the pH and rendered the fruit beverage less acidic and hence less erosive.

The other possible mechanism to explain the lesser erosive nature of the fortified fruit beverage could be that the chelation of the calcium to the acidic ions present in the beverage is correlated to reducing the active acid content and limiting the pH drop, thereby reducing the erosive potential of the beverage fortified with calcium ions [21].

Calcium ion concentration in the beverage also strongly influences the erosion rate. A higher degree of saturation of calcium ions lowers the rate at which erosion of the surface occurs [13]. Calcium present in the fruit beverages raises the degree of saturation in the Nernst layer, thereby enhancing the number of calcium and phosphate ions diffusing from the tooth surface [23, 25]. In our study, the calcium ion concentration in the fortified fruit beverage group reduced from baseline to 1 and 2 min after exposure, which means that calcium ions depleted from the fruit beverage and were now present in the Nernst layer around the tooth, thereby reducing the dissolution rate of enamel. As the Nernst layer becomes supersaturated with calcium, the calcium ions now leach out of the tooth into the fortified fruit beverage. Hence, the calcium concentration increased from 2 to 3 min after exposure.

The calcium ion concentration in the nonfortified fruit beverage group increased from baseline to 1 min after exposure, significantly suggesting loss of calcium ions from tooth structure in the beverage. The calcium ion concentration reduced from 1 to 2 min after exposure. This may be due to the supersaturation of calcium ions in fruit beverages with respect to the tooth leading to the movement of calcium ions back into the tooth surface. Similarly, the calcium ion concentration remained in supersaturation in fruit beverages from 2 to 3 min of exposure, suggesting that no further loss of calcium ions took place from the teeth samples after 1 min. Hence, it can be concluded that the nonfortified fruit beverage can be more erosive within the first 1 min of exposure. The results of our study are in agreement with other studies conducted on similar fruit beverages [11, 13].

Surface profilometry quantifies the loss of dental tissue in a nontreated reference area. It provides information on surface roughness-most commonly Ra (arithmetic average of surface roughness) and Rz (mean of 5 roughness depths). It can be used to measure erosion depth on natural surfaces [22]. The samples in the fortified fruit beverage group showed no significant change in the Ra from baseline to 10, 20, and 30 min after exposure. The Rz value showed a significant difference at 20 and 30 min compared to baseline. The nonfortified group showed a substantial increase in the Ra and Rz values after exposure at 30 min. This suggests that significant change in surface roughness can be observed only after prolonged exposure of samples to erosive beverages. These observations are in agreement with the results of the study conducted by Hara and Zero [18].

Microhardness of the enamel surface has been used to indicate changes in mineral density and monitor the early stages of enamel dissolution. The samples from the fortified fruit beverage group showed a significant increase in microhardness from baseline to 10, 20, and 30 min after exposure. Fortification of fruit beverages with calcium improves the physical properties of the tooth, such as microhardness. The increased level of calcium ions saturates the tooth surface, thereby preventing their dissolution. The samples in the nonfortified fruit group showed a significant decrease in hardness number from baseline to 10, 20, and 30 min after exposure suggestive of erosion. This suggests that exposure to fruit beverages with higher calcium ion concentration prevents the dissolution of the tooth's surface. The study conducted by Scaramucci et al. evaluating the surface hardness of teeth samples exposed to fruit beverages with higher pH and calcium ions obtained similar results [26].

In our study, fortification of fruit beverage with calcium ions (Tropicana Orange Delight) demonstrated a lesser erosive effect on primary teeth compared to the nonfortified fruit beverage with respect to the pH and calcium ion concentration of the beverage; the prolonged exposure of teeth samples to fortified fruit beverage did not show a significant change in surface roughness and microhardness. The samples exposed to nonfortified fruit beverages demonstrated a significant change in surface roughness and microhardness of the tooth. This suggests that the calcium ion fortification of fruit beverages causes significantly less degree of erosion on the tooth surface due to repeated or long-term exposure.

Based on the results of the present study, the null hypothesis, “There is no difference in the erosive potential of fortified and nonfortified fruit beverages on primary teeth,” was rejected.

In this in vitro study, artificial saliva was used, and demineralisation-remineralisation cycles that occur in in vivo conditions were also not simulated. These are the limitations in this study. Dental erosion is a dynamic process that results from interplay of protective factors and causative factors [26, 27]. Salivary proteins, pellicle, use of remineralising dentifrices, individual consumption practices, and so on play a significant role in the degree of erosive changes that occur in the oral cavity. All of these factors cannot be simulated in vitro studies evaluating dental erosion. Hence, the results of in vitro study cannot be exactly extrapolated to clinical situations.

## 5. Conclusion

The present study shows that fortification of fruit beverages with calcium ions is a successful alternative to render a beverage less erosive as compared to the nonfortified beverage. Unusual drinking practices can irreversibly damage the tooth due to extended periods of exposure; however, calcium-fortified fruit beverages offer significant protection against erosion in these cases.

## Figures and Tables

**Table 1:** Inter- and intragroup comparisons of pH and calcium ion concentration in groups A and B.

Duration of exposure	Group A		Group B		*p*-value^†^
	Mean	SD	Mean	SD	
pH					
Baseline^a^	6.96	0.10	3.92	0.09	<0.001
1 min^b^	6.87	0.14	3.63	0.06	<0.001
2 min^c^	6.94	0.07	3.64	0.07	<0.001
3 min^d^	6.86	0.08	3.63	0.08	<0.001
*p*-value^‡^	0.002		<0.001		
Post hoc test	a > b, d		a > b, c, d		

Calcium ion concentration (mmol/L)					
Baseline^a^	7.88	0.64	4.16	0.37	<0.001
1 min^b^	5.29	0.4	7.17	0.45	<0.001
2 min^c^	5.77	0.33	4.99	0.40	<0.001
3 min^d^	6.48	0.46	4.28	0.87	<0.001
*p*-value^‡^	<0.001		<0.001		
Post hoc test	a > d > b, c		a, c, d		

*Note.* ^‡^ *p*-value for intragroup comparisons and ^†^*p*-value for intergroup comparisons. An independent sample *t*-test was used for intergroup comparison and repeated measures ANOVA with post hoc Bonferroni test was used for intragroup comparison.

**Table 2:** Inter- and intragroup comparison of Ra (arithmetic average of surface roughness), Rz, and microhardness in groups C and D.

Duration of exposure	Group C		Group D		*p*-value^†^
	Mean	SD	Mean	SD	
Ra value (*μ*m)					
Baseline^a^	6.29	0.55	4.82	0.71	<0.001
10 min^b^	6.13	0.43	5.66	1.06	0.209
20 min^c^	5.97	0.81	5.99	1.05	0.953
30 min^d^	5.67	0.54	6.23	1.04	0.15
*p*-value^‡^	0.115		0.001		
Post hoc test	—		c, d > a		

Rz values (*μ*m)					
Baseline^a^	22.12	2.40	17.03	2.39	<0.001
10 min^b^	20.80	1.21	19.42	3.75	0.291
20 min^c^	20.30	2.00	19.98	3.63	0.811
30 min^d^	19.74	1.46	22.39	4.99	0.136
*p*-value^‡^	0.005		<0.001		
Post hoc test	a > c, d		d > a		

Microhardness (HV/10)					
Baseline^a^	345.61	7.94	343.58	5.22	0.509
10 min^b^	355.54	10.53	336.02	5.88	<0.001
20 min^c^	358.21	11.15	332.12	4.53	<0.001
30 min^d^	359.44	12.36	327.58	3.11	<0.001
*p*-value^‡^	<0.001		<0.001		
Post hoc test	b, c, d > a		a > b > c, d		

*Note.* Ra: arithmetic average of surface roughness, Rz: mean of 5 surface roughness, ^‡^*p*-value for intragroup comparisons, and ^†^*p*-value for intergroup comparisons.

## Data Availability

The data set of the study is available on request from Deepika Pai (deepikapai0479@gmail.com).

## Abbreviations

CaCo3: Calcium carbonate
Ra: Arithmetic average of surface roughness
Rz: Mean of 5 surface roughness.

## References

[1] Kanzow P., Wegehaupt F. J., Attin T., Wiegand A. (2016). Etiology and pathogenesis of dental erosion. *Quintessence International*.

[2] Tao D. Y., Hao G., Lu H., Tian Y., Feng X. P. (2015). Dental erosion among children aged 3–6 years and its associated indicators. *Journal of Public Health Dentistry*.

[3] Harding M. A., Whelton H., O’Mullane D. M., Cronin M. (2003). Dental erosion in 5-year-old Irish school children and associated factors: a pilot study. *Community Dental Health*.

[4] Aguiar Y. P. C., Santos F. G. D. F., Moura E. F. D. F. (2014). Association between dental erosion and diet in Brazilian adolescents aged from 15 to 19: a population-based study. *The Scientific World Journal*.

[5] Schlueter N., Luka B. (2018). Erosive tooth wear—a review on global prevalence and on its prevalence in risk groups. *British Dental Journal*.

[6] Corica A., Caprioglio A. (2014). Meta-analysis of the prevalence of tooth wear in primary dentition. *European Journal of Paediatric Dentistry*.

[7] Gatt G., Attard N. (2019). Erosive wear of the primary dentition: who is aware of it?. *European Archives of Paediatric Dentistry*.

[8] De Menezes Oliveira M. A. H., Torres C. P., Gomes-Silva J. M. (2010). Microstructure and mineral composition of dental enamel of permanent and deciduous teeth. *Microscopy Research and Technique*.

[9] Attin T., Weiss K., Becker K., Buchalla W., Wiegand A. (2005). Impact of modified acidic soft drinks on enamel erosion. *Oral Diseases*.

[10] Beiraghi S., Atkins S., Rosen S., Wilson S., odom J., Beck M. (1989). Effect of calcium lactate in erosion and S. mutans in rats when added to Coca‐Cola. *Pediatric Dentistry*.

[11] Barbour M. E., Parker D. M., Allen G. C., Jandt K. D. (2005). Human enamel erosion in constant composition citric acid solutions as a function of degree of saturation with respect to hydroxyapatite. *Journal of Oral Rehabilitation*.

[12] Larsen M. J., Nyvad B. (1999). Enamel erosion by some soft drinks and orange juices relative to their pH, buffering effect and contents of calcium phosphate. *Caries Research*.

[13] Stefanski T., Postek-Stefańska L. (2014). Possible ways of reducing dental erosive potential of acidic beverages. *Australian Dental Journal*.

[14] Ehlen L. A., Marshall T. A., Qian F., Wefel J. S., Warren J. J. (2008). Acidic beverages increase the risk of in vitro tooth erosion. *Nutrition Research*.

[15] Eisenburger M., Addy M. (2003). Influence of liquid temperature and flow rate on enamel erosion and surface softening. *Journal of Oral Rehabilitation*.

[16] Ali H., Tahmassebi J. F. (2014). The effects of smoothies on enamel erosion:an in situ study. *International Journal of Paediatric Dentistry*.

[17] Allen L., Benoist B., Dary O., Hurrell R. (2006). *Guidelines on food fortification with micronutrients. France*.

[18] Hara A. T., Zero D. T. (2008). Analysis of the erosive potential of calcium-containing acidic beverages. *European Journal of Oral Sciences*.

[19] Davidson C. L., Hoekstra I. S., Arends J. (1974). Microhardness of sound, decalcified and etched tooth enamel related to the calcium content. *Caries Research*.

[20] Chuenarrom C., Benjakul P., Daosodsai P. (2009). Effect of indentation load and time on knoop and vickers microhardness tests for enamel and dentin. *Materials Research*.

[21] Franklin S., Masih S., Thomas A. M. (2014). An in-vitro assessment of erosive potential of a calcium-fortified fruit juice. *European Archives of Paediatric Dentistry*.

[22] Lussi A., Jaeggi T. (2006). Dental erosion Monographs in oral science. *Chemical Factors*.

[23] Seow W. K., Thong K. M. (2005). Erosive effects of common beverages on extracted premolar teeth. *Australian Dental Journal*.

[24] Meurman J. H., Harkonen M., Naveri H. (1990). Experimental sports drinks with minimal dental erosion effect. *European Journal of Oral Sciences*.

[25] Barbour M. E., Lussi A., Shellis R. P. (2011). Screening and prediction of erosive potential. *Caries Research*.

[26] Scaramucci T., Hara A. T., Zero D. T., Ferreira S. S., Aoki I. V., Sobral M. A. P. (2011). Development of an orange juice surrogate for the study of dental erosion. *Brazilian Dental Journal*.

[27] Wegehaupt F. J., Attin T. (2010). The role of fluoride and casein phosphopeptide/amorphous calcium phosphate in the prevention of erosive/abrasive wear in an in vitro model using hydrochloric acid. *Caries Research*.

